# Empowering Ethiopia’s digital citizenship in early-career healthcare leadership

**DOI:** 10.1371/journal.pdig.0000845

**Published:** 2025-05-07

**Authors:** Sharmi Haque

**Affiliations:** 1 Faculty of Health, Medicine and Life Sciences, Maastricht University, Maastricht, The Netherlands,; 2 Medics.Academy, London, United Kingdom; McGill University, CANADA

Digital citizenship is an integral part of healthcare leadership and extends beyond technical competence. For young healthcare professionals (HCPs), it denotes the ability to responsibly participate with digital tools, data, governance, and policy in health, digital, and civic engagement [1]. As Ethiopia undertakes digital transformation in healthcare, digital health citizenship is becoming a vital competency for HCPs [2]. In 2022–2023, only 56% of Ethiopian HCPs demonstrated high digital health literacy [3]. Contemporary healthcare leadership extends beyond clinical expertise, encompassing digital literacy, critical evaluation of clinical data, and proactive participation in policy [4]. Without these skills, aspiring HCP leaders will find it challenging to navigate the contemporary digital landscape [5].

Ethiopian healthcare leadership demands good digital health literacy to effectively implement digital transformation [6]. Ethiopia’s Digital Health Blueprint places a focus on the need for better digital transformation applications, especially in clinical data management and health applications [7]. Limited technical proficiency among young HCPs delays engagement with digital tools, increasing the risk of falling behind in digital health adoption and transformation [8]. Bridging Ethiopia’s digital divide requires strong health literacy, allowing HCPs to align with digital health developments that are responsive to community needs, particularly in rural areas [9].

Initiatives such as M-Health Ethiopia require HCPs to interpret digital data, educate populations, and implement public-informed interventions [10]. Digital solutions may be underutilized or misinterpreted without health literacy [11]. Young HCPs have had limited involvement in health policy decision-making; active participation in digital health governance presents opportunities for inclusive and sustainable policy implementation [12]. Projects like Ethiopian Digital Health Innovation and Entrepreneurship empower emerging healthcare leaders to engage in policy advocacy for equitable digital health access [13].

Ethiopia demonstrates both digital engagement and leadership among young HCPs through initiatives like the Leadership Incubation Program for Health, which prepares young HCPs to influence policy through healthcare leadership and strategic decision-making [14]. The Digital Health Activity actively supports youth-driven digital health innovation by providing mentorship, funding, and education to young HCPs in developing digital health solutions through digital entrepreneurship [15]. Training in digital health competencies and data-driven decision-making showcases how increasing access to digital health citizenship can improve Ethiopia’s healthcare leadership pipeline [6].

Ethiopia’s Digital Health Blueprint aims to engage young HCPs in policymaking to drive innovative and equitable healthcare transformation [7]. Its central focus revolves around engaging young HCPs in health policy, governance, and digital innovation [8]. It is paramount that young HCPs actively contribute to and implement health policy that is responsive to public and local needs and further influence health policies through governance structures [9]. The International Institute for Primary Health Care-Ethiopia (IPHC-E) has evidenced that HCPs engaged in policy advocacy and leadership have enabled capacity building in digital health tools, strengthening digital governance, and improving health service delivery [10]. Investing in continual digital health literacy training can accelerate young HCPs’ acquisition of skills necessary for emerging digital health tools [11]. By embedding greater involvement of young HCPs in digital health policymaking, Ethiopia can cultivate informed, proactive healthcare leaders to drive sustainable healthcare innovation [12].

Digital health leadership demands inclusivity, particularly gender representation in decision-making [13]. Women comprise a substantial proportion of Ethiopia’s healthcare workforce, but remain underrepresented in digital healthcare leadership [14]. Gender-inclusive leadership is necessary to provide a holistic approach to digital health governance [15]. Partnerships such as Medics.Academy, Ethiopian Medical Women’s Association, and Mekelle University, highlight how female HCPs are supported through digital healthcare leadership training [6]. Support for initiatives like this from the Ethiopian Ministry of Health, aims to provide digital training to 70% of female doctors over five years to address the workforce crisis [7]. It emphasizes the instrumental value of building digital healthcare leadership in professional development [8]. The Higher Education Readiness Programme is an initiative that focuses on equipping young females with the necessary skills and confidence to excel in digital health [9]. Through mentorship programs and inclusive opportunities, it facilitates the active engagement of females in digital health [10]. Closing the gender gap in digital health expansion requires leadership training [11]. Ethiopia can inspire female HCPs to drive digital health transformation and lead policy development [12].

Significant concerns have been raised about Ethiopia’s increasing reliance on AI-driven health solutions, particularly regarding digital platforms, ethical governance, and data privacy [13]. Digital health citizenship ensures ethical data governance and patient safety amid rapid healthcare digitalization [14]. In the absence of robust cybersecurity, AI ethics, and data protection, risks such as data breaches, privacy violations, and algorithmic bias in AI can compromise both patient trust and public health [15]. Initiatives like The DigHealth Project (Erasmus+) provide training in digital ethics, cyber-governance, and health data protection to inform young HCPs on responsible digital health technology usage [6]. Digital Platform 8335 is a trusted source combating misinformation by providing evidence-based medical knowledge to professionals and the public [7].

To bolster digital health citizenship in Ethiopia, key policy reforms are necessary [8]. Recommendations include integrating digital health literacy training within medical education, increasing youth participation in health governance, incorporating digital health literacy into university curricula, and fostering partnerships between healthcare, educational, and digital health initiatives [9]. These strategies will equip young HCPs with the skills to drive digital transformation and shape the future of Ethiopia’s healthcare system [10].
